# Endemic Radiation of African Moonfish, *Selene dorsalis* (Gill 1863), in the Eastern Atlantic: Mitogenomic Characterization and Phylogenetic Implications of Carangids (Teleostei: Carangiformes)

**DOI:** 10.3390/biom14101208

**Published:** 2024-09-25

**Authors:** Emmanuel Ofosu Mireku Ewusi, Soo Rin Lee, Ah Ran Kim, Yunji Go, Hsu Htoo, Sangdeok Chung, Muhammad Hilman Fu’adil Amin, Sapto Andriyono, Hyun-Woo Kim, Shantanu Kundu

**Affiliations:** 1 Department of Marine Biology, Pukyong National University, Busan 48513, Republic of Korea; 2 Fisheries Commission, Ministry of Fisheries and Aquaculture Development, Fisheries Scientific Survey Division, Tema P.O. Box BT 62, Ghana; 3 Research Center for Marine Integrated Bionics Technology, Pukyong National University, Busan 48513, Republic of Korea; 4 Marine Integrated Biomedical Technology Center, National Key Research Institutes in Universities, Pukyong National University, Busan 48513, Republic of Korea; 5 Industry 4.0 Convergence Bionics Engineering, Pukyong National University, Busan 48513, Republic of Korea; 6 Distant Water Fisheries Resources Research Division, National Institute of Fisheries Science, Busan 46083, Republic of Korea; 7 Advanced Tropical Biodiversity, Genomics, and Conservation Research Group, Department of Biology, Faculty of Science and Technology, Airlangga University, Surabaya 60115, Indonesia; 8 Department of Marine, Faculty of Fisheries and Marine, Airlangga University, Surabaya 60115, Indonesia; 9 Ocean and Fisheries Development International Cooperation Institute, College of Fisheries Science, Pukyong National University, Busan 48513, Republic of Korea; 10 International Graduate Program of Fisheries Science, Pukyong National University, Busan 48513, Republic of Korea

**Keywords:** Atlantic Ocean, marine fish, mitochondrial genome, evolutionary relationship, lineage diversification, conservation

## Abstract

This study offers an in-depth analysis of the mitochondrial genome of *Selene dorsalis* (Gill 1863), a species native to the Eastern Atlantic Ocean. The circular mitochondrial DNA molecule measures 16,541 base pairs and comprises 13 protein-coding genes (PCGs), 22 transfer RNA (tRNA) genes, two ribosomal RNA genes, and a control region (CR). The nucleotide composition exhibits a notable adenine-thymine (AT) bias, accounting for 53.13%, which aligns with other species in the Carangidae family. Most PCGs initiate with the ATG codon, with the exception of Cytochrome C oxidase subunit I, which starts with GTG. Analysis of relative synonymous codon usage reveals that leucine and serine are the most prevalent amino acids in the mitochondrial genome of *S. dorsalis* and its congeners (*S. vomer* and *S. setapinnis*). All tRNAs display the typical cloverleaf structure, though *tRNA Serine* (S1) lacks a dihydrouracil arm. Pairwise comparisons of synonymous and nonsynonymous substitutions for all PCGs yielded values below ‘1’, indicating strong purifying selection. The CR spans 847 bp, representing 5.12% of the mitochondrial genome, and is characterized by high AT content (62.81%). It is situated between *tRNA-Pro* (TGG) and *tRNA-Phe* (GAA). The CR contains conserved sequence blocks, with CSB-1 being the longest at 22 bp and CSB-D the shortest at 18 bp. Phylogenetic analysis, using Bayesian and Maximum-likelihood trees constructed from concatenated PCGs across 72 species, successfully differentiates *S. dorsalis* from other carangids. This study also explores how ocean currents and gyres might influence lineage diversification and parapatric speciation of *Selene* species between the Atlantic and Pacific Oceans. These results highlight the importance of the mitochondrial genome in elucidating the structural organization and evolutionary dynamics of *S. dorsalis* and its relatives within marine ecosystems.

## 1. Introduction

The mitochondrial genome plays a vital role in all eukaryotic organisms, providing insights into ancient and matrilineal evolutionary relationships [1]. In vertebrates, the mitochondrial genome is highly compact, typically spanning 16 to 17 kilobase pairs, and exhibits remarkable conservation in the array of genes it harbors [2]. The mitogenome predominantly encodes 13 protein-coding genes (PCGs), two ribosomal RNAs (rRNAs), and 22 transfer RNAs (tRNAs), along with one noncoding region known as the control region (CR). Additionally, mitochondrial genes exhibit highly conserved genetic traits across different taxa, particularly among bilaterian metazoans, showing unique similarities in both size and base composition [3]. These genes are crucial components of the eukaryotic cell’s genetic arsenal, making them invaluable for studying maternal lineages and evolutionary patterns [4]. Therefore, it is essential to elucidate the configuration and variability of the mitogenome in each organism to understand its functionalities and potential mutations on the L-strand or H-strand, which are most likely to arise from base substitutions [5]. Moreover, beyond mitochondrial and nuclear partial genes, complete mitochondrial genomes have proven successful in various aspects of biodiversity research including ichthyology [6,7,8].

Carangid fishes (Carangiformes: Carangidae), commonly referred to as jacks, trevallies, scads, amberjacks, queen fishes, runners, pilot fish, and pompano, are widely distributed across tropical and subtropical marine ecosystems [9]. These coastal pelagic fishes hold significant economic importance on a global scale [10]. This taxonomic order includes a total of 1103 valid species under 198 genera worldwide, with the most species-rich families being Bothidae (169 species), Carangidae (153 species), Cynoglossidae (168 species), and Soleidae (179 species). The family Carangidae is represented by 39 genera within four subfamilies: Caranginae, Naucratinae, Scomberoidinae, and Trachinotinae [11]. Among these, the subfamily Caranginae comprises 104 valid species across 29 genera, including the distinctive moonfish genus *Selene*, which contains six valid species distributed globally. Of these, five species (*Selene brevoortii*, *Selene brownii*, *Selene peruviana*, *Selene setapinnis*, and *Selene vomer*) are found in the Eastern Pacific and Western Atlantic oceans. However, a single species, the African moonfish (*Selene dorsalis*), is distributed from the Eastern Atlantic to the Southwestern Mediterranean Sea [12].

The African moonfish (*S. dorsalis*) is an economically significant species native to the Atlantic Ocean, ranging from southern Portugal to South Africa, including Madeira, the Cape Verde Islands, and São Tomé and Principe [13]. Commonly referred to as lookdowns or moonfish, these fish are characterized by their distinctive head profile, eye placement, horizontally compressed deep bodies, and circular profiles [12]. This unique morphology is shared among other *Selene* species within Carangidae [14]. Beyond its native range, *S. dorsalis* has also been reported in the Central Mediterranean Sea near Munxar Reef, located in the shallow waters off the southeastern coastline of the island of Malta, and in the Canary Islands [12,15,16,17,18]. This unusual distribution may result from the species’ introduction or migration into colder regions, potentially driven by rising ocean temperatures in the Gulf of Guinea [19]. According to the United Nations Food and Agriculture Organization (FAO), the African moonfish is a common component of the ichthyofauna in the Gulf of Guinea and is highly sought after in Ghana, where it contributes to an annual per capita fish consumption of 25 kg [9]. Additionally, recent studies estimate that *Selene dorsalis* is heavily harvested within tropical fishing zones [9]. Despite significant anthropogenic pressures, the International Union for Conservation of Nature (IUCN) Red List categorizes all *Selene* species as being of ‘Least Concern’ [20].

Remarkably, most studies on the African moonfish primarily focused on its biology, ecology, and taxonomy [9,12,21]. Given that genetic and physiological changes in fish are closely tied to their ecological significance, obtaining genetic information from both native and extended ranges of any fish species is essential [22,23]. In this context, it is crucial to acquire and thoroughly analyze genetic data, such as the mitochondrial genome of species endemic to specific geographical locations, to provide insights into taxonomic classification, evolutionary history, and genetic traits. Such information is vital for understanding population structure and establishing effective management policies [6,24]. Within Carangidae, molecular studies have been particularly important due to unstable morphological characteristics and frequent taxonomic revisions [25].

The initiative to generate molecular data for *Selene* species began long ago for various scientific purposes. The mitochondrial *CYTB* gene of *Selene dorsalis* was sequenced to aid in the systematic classification of *Selene* species through cladistic analyses [14]. Additionally, a new nuclear marker (*RNF213*) and multi-gene-based investigations were initiated to elucidate the phylogeny of acanthomorphs and the taxonomic placement of carangids, including *S. dorsalis* [26,27]. To enhance the comprehensive application of the regulatory Fish Encyclopedia, identify early life stages for fisheries management, and track ornamental fishes, the mitochondrial DNA barcoding region of *Selene* species was also sequenced [28,29,30]. Furthermore, partial mitochondrial genes were sequenced for *Selene* species to assess genetic diversity across diverse marine ecosystems, such as São Paulo State in Brazil, Caribbean reefs, and the North Atlantic Ocean [31,32,33]. Moreover, to elucidate multi-locus phylogeny and build the tree of life for confirming the new classification of bony fishes, *Selene* species’ gene sequences were incorporated into previous research to clarify evolutionary relationships and diversification [34,35,36]. Beyond systematics and evolutionary research, DNA sequences of *Selene* species were also generated to build a reference standard sequence library for DNA-based commercial fish and seafood identification, prey–predator relationship studies, and metabarcoding-based environmental DNA research [37,38,39,40,41,42,43].

In recent years, the complete mitochondrial genomes of two *Selene* species (*S. setapinnis* and *S. vomer*) have been sequenced [44,45]. However, previous studies have not provided a detailed analysis of the structure and variations within these *Selene* mitogenomes, which is essential for understanding gene features, structural variability, and conducting in-depth maternal phylogenetic analyses [46,47,48]. Additionally, both species whose mitochondrial genomes were sequenced are distributed in the Western Atlantic, including the Gulf of Mexico and the Caribbean Sea. This leaves a significant research gap in understanding the evolutionary relationships and diversification patterns of their Eastern Atlantic congener, *S. dorsalis*. Therefore, the aim of this study was to generate a novel mitochondrial genome for *S. dorsalis* and to characterize its structure and variability as well as cladistic analyses. This will enhance our understanding of its unique genetic traits, biogeographic evolution, maternal lineage diversification, and subsequent adaptation in the Eastern Atlantic Ocean. This study will reveal the mitogenomic signature of the species in the Eastern Atlantic and facilitate the use of multiple mitochondrial markers for species identification and population structure analysis, crucial for conserving ecologically and economically important regional species.

## 2. Materials and Methods

### 2.1. Sample Collection, Identification, and Preservation

**Figure 1:**
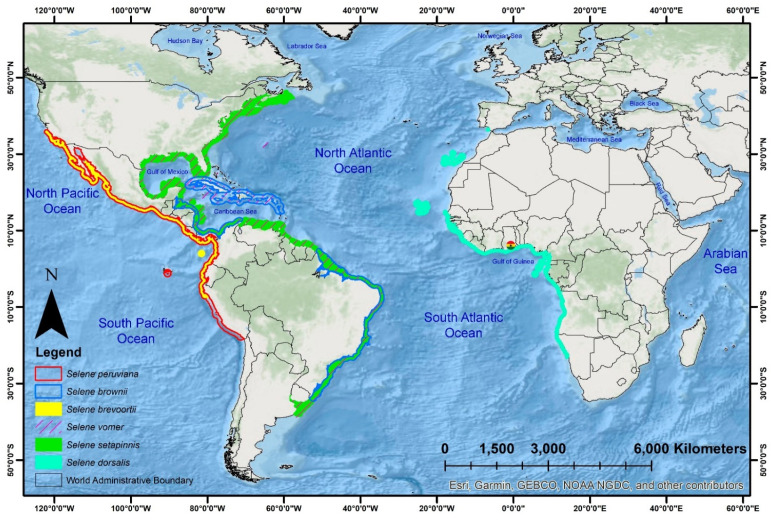
Global distribution pattern of *Selene* species. The collection locality for *S. dorsalis* is indicated by the round-shaped country map of Ghana. The map was generated using ArcGIS version 10.6 and manually edited in Adobe Photoshop CS 8.0.

Among multiple catches, a single specimen of moonfish was collected from the Atlantic Ocean at coordinates 5.611389 N, 0.044444 W on 22 February 2024 (Figure 1). The specimen was identified as *S. dorsalis* using taxonomic keys described in previous studies and was preserved with the novel voucher number ‘GH1’ at the Fisheries Scientific Survey Division of Ghana [25,49]. Under aseptic conditions, approximately 20 g of tissue were extracted from the apexial muscle, preserved in 95% molecular-grade ethanol in a 2 mL centrifuge tube, and stored in a −20 °C freezer. To minimize DNA degradation and prevent microbial contamination, the tissue sample in the centrifuge tube was tightly sealed with Parafilm and transported in an icebox to maintain optimal temperature conditions. It was then sent to the Molecular Physiology Laboratory at Pukyong National University, Republic of Korea, for further molecular experiments. Distribution data for *S. dorsalis* and other *Selene* species were obtained from the IUCN (https://www.iucnredlist.org/ accessed on 15 August 2024) and mapped to understand their unique biogeographic distribution in both the Eastern and Western Atlantic and Pacific Oceans (Figure 1). The fish specimen was caught by the local fishing community, and the deceased individual was collected by the researcher; therefore, no animal ethics approval was required for the biological sample collection. Furthermore, the molecular data generation and analyses were approved by Pukyong National University (PKNUIACUC-2022-72), ensuring that the use of biological material in the experiments adhered to ethical standards.

### 2.2. Genomic DNA Extraction and Partial Gene Sequencing

The AccuPrep^®^ Genomic DNA Extraction Kit (Bioneer, Daejeon, Republic of Korea) was used to extract genomic DNA according to the manufacturer’s standard protocols. The quality of the extracted DNA was assessed using a NanoDrop spectrophotometer (Thermo Fisher Scientific, D1000, Waltham, MA, USA). Specifically, 30 mg of tissue from the target specimen was homogenized in 600 μL of 1× lysis buffer using a Tissue Lyser II (Qiagen, Hilden, Germany) for 60 s. Sodium dodecyl sulfate (SDS) (100 μL) and the proteolytic enzyme proteinase K (20 μL) were then added to disrupt cell membranes and degrade proteins. The mixture was incubated at 60 °C for 12 h. Following this, 500 μL of GC buffer and 300 μL of isopropanol were added to facilitate DNA precipitation. The resulting solution was transferred to a column tube and centrifuged at 8000 rpm for one minute. Washing buffers 1 and 2 were used to remove any residual biomolecular substances, and finally, 50 μL of TE buffer was used to elute the target DNA.

Following DNA extraction, polymerase chain reaction (PCR) was performed using the universal primers Fish-BCH (5′-TCAACYAATCAYAAAGATATYGGCAC-3′) and Fish-BCL (5′-ACTTCYGGGTGRCCRAARAATCA-3′) to amplify a partial sequence of the mitochondrial *COI* gene for preliminary DNA sequence-based species identification [50]. PCR was carried out using a Takara thermal cycler with a 30 µL reaction mixture consisting of 1 μL each of forward and reverse primers, 0.9 μL of 3% dimethyl sulfoxide (DMSO), 19.9 μL of sterilized deionized water, 3 μL of 10× ExTaq Buffer, 0.2 μL of Ex Taq HS enzyme, 3 μL of dNTPs, and 1 μL of 1/10 diluted target DNA template. The thermal cycling conditions included an initial denaturation at 94 °C for 3 min, followed by 40 cycles of denaturation at 94 °C for 30 s, annealing at 50 °C for 30 s, extension at 72 °C for 1 min, and a final extension at 72 °C for 5 min. The PCR product was purified using the AccuPrep^®^ PCR/Gel Purification Kit (Bioneer, Republic of Korea) and sequenced bidirectionally with a 96-capillary automated ABI PRISM 3730XL Analyzer at Macrogen (https://dna.macrogen.com/, Daejeon, Republic of Korea). Noisy regions in the bidirectional chromatogram results were removed using SeqScanner version 1.0 (Applied Biosystems Inc., Foster City, CA, USA). The resulting *COI* sequences were analyzed and confirmed through a nucleotide BLAST search (https://blast.ncbi.nlm.nih.gov accessed on 15 August 2024) against the global GenBank database.

### 2.3. Mitogenome Sequencing and Assembly

To obtain the complete mitogenome of *S. dorsalis*, paired-end (2 × 150 bp) next-generation sequencing (NGS) was performed on the NovaSeq platform at Macrogen (Illumina, Inc., San Diego, CA, USA). Sequencing libraries were prepared according to the manufacturer’s specifications for the TruSeq Nano DNA High-Throughput Library Prep Kit (Illumina, Inc., San Diego, CA, USA). A total of 100 ng of genomic DNA was fragmented using adaptive focused acoustic technology (Covaris, Woburn, MA, USA), resulting in double-stranded DNA molecules with blunt ends and 5′-phosphorylation. After end-repair, DNA fragments were size-selected using a bead-based method, modified with the addition of a single ‘A’ base, and ligated with TruSeq DNA UD Indexing adapters. The library was then purified and enriched through PCR to produce the final DNA library. Library quantification was performed using qPCR following the qPCR Quantification Protocol Guide (KAPA Library Quantification Kits for Illumina Sequencing Platforms), and quality assessment was conducted using an Agilent Technologies 4200 TapeStation D1000 screentape (Agilent Technologies, Santa Clara, CA, USA).

High-quality NGS sequences were assembled using Geneious Prime v2023.0.1 and mapped against the reference mitogenome (*S. vomer* Accession No. PP033011) [51]. To verify PCGs, overlapping regions were aligned using MEGA X software. The boundaries and directions of other genes were confirmed using the MITOS Galaxy web server (http://mitos.bioinf.uni-leipzig.de accessed on 15 August 2024) and MitoAnnotator (http://mitofish.aori.u-tokyo.ac.jp/annotation/input/ accessed on 15 August 2024) [52,53,54]. Additionally, the boundaries of each PCG were further validated through Open Reading Frame Finder (https://www.ncbi.nlm.nih.gov/orffinder/ accessed on 15 August 2024) after translation into the respective amino acids. The final mitogenome of *S. dorsalis* was submitted to the GenBank database.

### 2.4. Characterization and Comparative Analyses

To generate a three-dimensional representation of the mitogenome, MitoAnnotator was employed. The primary objective of the analysis was to fully characterize the mitogenome and to identify significant variations compared to the existing mitogenomes of two other *Selene* species (*S. vomer*: PP033011 and *S. setapinnis*: OR575618). Intergenic spacers between contiguous genes and overlapping regions were manually calculated. The nucleotide compositions of 13 PCGs, two rRNAs, 22 tRNAs, and the CR were determined using MEGA X.

Nucleotide diversity (π) was assessed using a sliding window approach with a window size of 200 bp and a step size of 25 bp, performed in DnaSP6.0 [55]. Base composition skews were calculated using the formulas AT-skew = [A − T]/[A + T] and GC-skew = [G − C]/[G + C] [56]. Additionally, the AT and GC skews, as well as codon saturation of the PCGs based on transitions (s) and transversions (v), were illustrated using DAMBE6 [57]. The initiation and termination codons for each PCG were determined according to the vertebrate mitochondrial genetic code using MEGA X in conjunction with MITOS. Further analyses included calculating the relative frequency of amino acids, relative synonymous codon usage (RSCU), and pairwise comparisons for synonymous (Ks) and nonsynonymous (Ka) substitutions between *S. dorsalis* and the two other *Selene* species using DnaSP6.0. The boundaries of rRNA and tRNA genes were validated using tRNAscan-SE Search Server 2.0 in combination with ARWEN 1.2 [58,59]. Structural domains in the control region were identified through CLUSTAL X alignments, as referenced in previous studies [46,60,61].

### 2.5. Dataset Preparation and Phylogenetic Analyses

To elucidate the matrilineal phylogenetic relationships within the Carangidae family, a dataset comprising 72 species mitogenomes (1 newly generated and 71 obtained from GenBank) representing four subfamilies was compiled (Table S1). The mitogenomes of two species from the family Coryphaenidae (*Coryphaena equiselis*, PP032965 and *Coryphaena hippurus*, OR582674); *Rachycentron canadum* (FJ154956) from the family Rachycentridae; *Nematistius pectoralis* (ON838225) from the family Nematistiidae; and four species from the family Echeneidae (*Remora albescens*, OP057074; *Remora brachyptera*, OR546234; *Remora osteochir*, OR575559; and *Echeneis naucrates*, AB355905) were designated as outgroups based on previous studies [62,63]. The iTaxoTools 0.1 was used to construct concatenated datasets of 13 PCGs to investigate the evolutionary relationships among Carangidae, with a particular focus on *Selene* species within the subfamily Caranginae [64]. Each PCG was analyzed separately to determine the optimal substitution model, which was found to be ‘GTR + G + I’ with the lowest Bayesian Information Criterion (BIC) scores, using PartitionFinder 2 and JModelTest v2 [65,66]. A Bayesian tree was constructed using MrBayes 3.1.2, which employs a Metropolis-coupled Markov chain Monte Carlo (MCMC) algorithm with nst = 6. The analysis was run for 10,000,000 generations, with samples collected every 100 generations, and 25% of the samples were discarded as burn-in [67]. The Maximum-Likelihood (ML) tree was further constructed with the ‘GTR + G + I’ model and default settings in PhyML 3.0 [68]. The resulting tree was visualized using the iTOL v4 web server for enhanced clarity [69].

## 3. Results and Discussion

### 3.1. Mitogenome Structure and Organization

**Figure 2:**
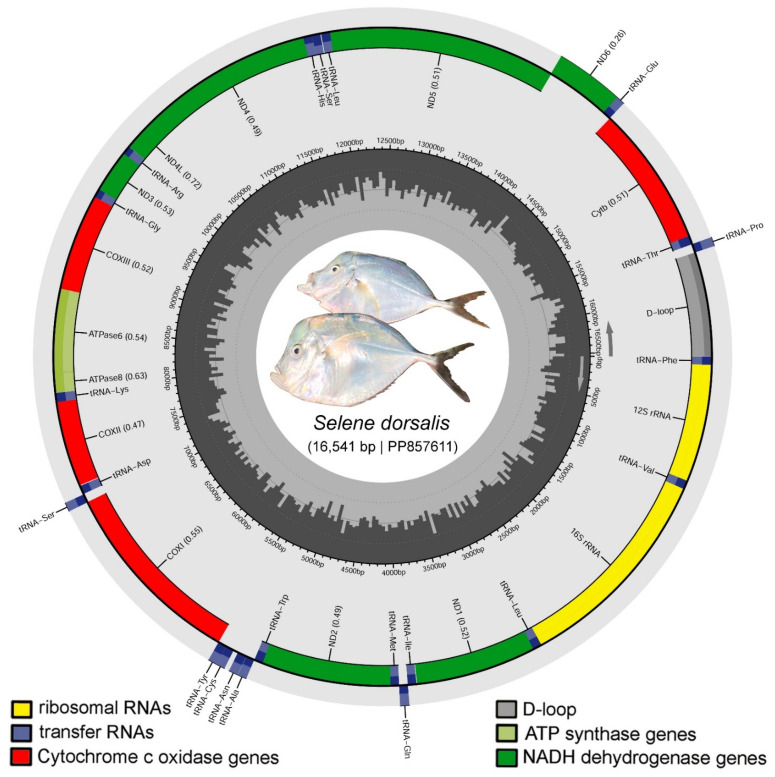
Circular mitochondrial genome of *S. dorsalis*, annotated using the MitoAnnotator online server. Different colored arcs represent the presence of PCGs, rRNAs, tRNAs, and CR. The species photograph was taken by the first author (E.O.M.E.).

In the present study, the mitogenome of *S. dorsalis* was characterized as being 16,541 bp long, with the GenBank accession number PP857611. This mitogenome includes 13 PCGs, 22 tRNAs, two rRNAs, and an AT-rich CR (Figure 2, Table 1). Notably, the *S. dorsalis* mitogenome is shorter than those of the other two *Selene* species (*S. vomer*: PP033011 and *S. setapinnis*: OR575618). The mitogenome of *S. dorsalis* exhibits a distinct gene arrangement, with 27 genes (12 PCGs, 2 rRNAs, and 14 tRNAs) located on the heavy strand, and a single PCG (*ND6*) and eight tRNAs (glutamine, proline, alanine, asparagine, cysteine, tyrosine, serine, and glutamic acid) on the light strand (Table 1). The mitogenome displays a notable AT-bias of 53.13%, with 27.48% adenine (A), 25.66% thymine (T), 30.18% cytosine (C), and 16.69% guanine (G). This AT-bias is also observed in the mitogenomes of *S. vomer* and *S. setapinnis*. The AT-skew and GC-skew for *S. dorsalis* were calculated as 0.034 and −0.288, respectively, while for *S. vomer*, they were 0.042 and −0.305 (Table 2). The mitogenome of *S. dorsalis* contains 19 overlapping regions totaling 40 bp in length, with the longest overlap (10 bp) occurring between *ATP6* and *ATP8*. Three overlapping PCG regions common to all examined *Selene* species were identified: 7 bp (*ND4L*), 4 bp (*ND5*), and 10 bp (*ATP8*). Additionally, the *tRNA-Asn* (N) gene has the longest intergenic spacer of 37 bp (Table S2). The variations observed in the mitogenomes of *Selene* species provide valuable insights into their evolutionary mechanisms, functional differences, and energy utilization, consistent with similar findings in other fish species [70]. This study enhances our understanding of the structural features of *Selene* mitogenomes and their associated genes.

### 3.2. Protein-Coding Genes

**Figure 3:**
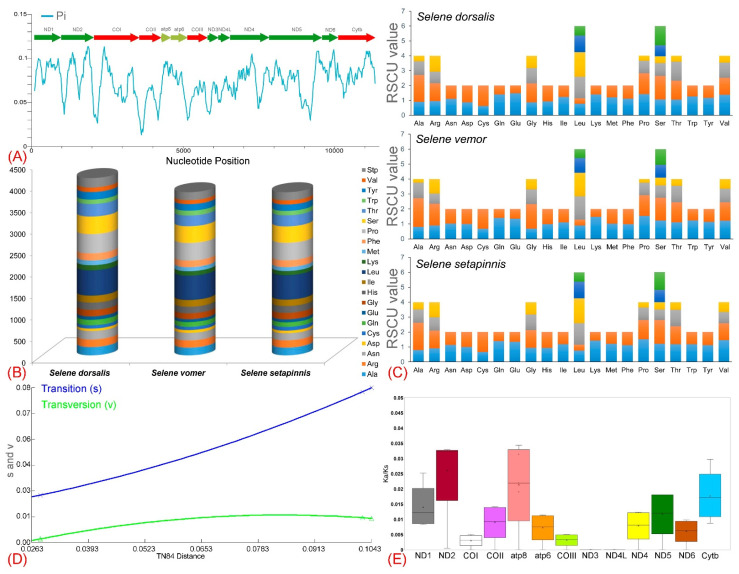
(**A**) Genetic diversity (Pi) of mitochondrial PCGs showing genetic variations among *S. dorsalis* and its congeners. (**B**) Codon usage abundance across the mitogenomes of three *Selene* species. (**C**) Comparative relative synonymous codon usage (RSCU) in *Selene* species, including *S. dorsalis*. (**D**) Substitution patterns in PCG matrices. The graph illustrates non-saturated trends in the variance of transitions and transversions as Kimura 2-parameter genetic distance increases. (**E**) Box plot depicting the pairwise divergence of Ka/Ks ratios for each mitochondrial PCG across all carangid fishes, including *Selene* species.

The mitogenome of *S. dorsalis* contains 13 PCGs with a combined length of 11,427 bp, which accounts for 69.10% of the total mitogenome. The shortest PCG is *ATP8* at 168 bp, while the longest is *ND5* at 1839 bp. Both *S. dorsalis* and *S. setapinnis* have PCG lengths of 11,427 bp, whereas *S. vomer* has slightly longer PCGs at 11,428 bp. Of the 13 PCGs, 12 in *S. dorsalis* use ATG (Methionine) as the initiation codon, while *COI* uses GTG. The typical termination codon TAA is observed in *ND1*, *ND4L*, *ND5*, *COI*, and *ATP8*. However, *ND2*, *ND3*, *ND4*, *COII*, *COIII*, *ATP6*, and *CYTB* have incomplete termination codons, which may be completed to TAA through polyadenylation during RNA maturation (Table S3) [71]. Comparative analysis of the PCGs across *Selene* species shows AT-skews ranging from −0.015 in *S. dorsalis*, −0.001 in *S. vomer*, to −0.037 in *S. setapinnis*. The GC-skew values range from −0.357 in *S. dorsalis*, −0.377 in *S. vomer*, to −0.326 in *S. setapinnis* (Table 2). Sliding window analysis of nucleotide diversity on concatenated PCGs yielded a nucleotide diversity value (Pi) of 0.07357 (Figure 3A). These genetic variations could contribute to independent selection pressures, positive selection, and evolutionary changes in the amino acids of PCGs [72,73,74]. These PCGs are crucial for encoding proteins involved in the electron transport chain, essential for oxidative phosphorylation and adenosine triphosphate (ATP) synthesis. Further studies on mitogenomes from additional *Selene* species could provide insights into variations in energy metabolism and protein expression profiles.

### 3.3. Codon Usage and Substitution Pattern

The amino acid utilization frequencies among the target species *S. dorsalis* and its congeners *S. vomer* and *S. setapinnis* were similar, with leucine, serine, and proline being the most abundant amino acids across all species. Conversely, aspartic acid, cysteine, and glutamic acid were the least abundant (Figure 3B). Analysis of the RSCU showed a total of 3912 codon transcriptions for *S. dorsalis*, 3616 for *S. vomer*, and 3611 for *S. setapinnis* (Table S4). For all three species, RSCU values were highest for leucine and serine compared to other amino acids, due to the involvement of six different coding nucleotides (Figure 3C). A saturation analysis indicated the non-saturated trends in the variance of transitions and transversions with an increasing Kimura 2-parameter genetic distance (Figure 3D). The calculation of the nonsynonymous (Ka) to synonymous (Ks) substitution ratio indicated that each PCG in the mitogenome of *S. dorsalis* and its related species within the Carangidae family is subject to similar selective pressures. Average pairwise Ka/Ks values ranged from a minimum of ‘0’ (*ND3* and *ND4L*) to a maximum of 0.0324 ± 0.00049 (*ND2*), following the order *ND3* < *ND4L* < *COI* < *COIII* < *ND6* < *ATP6* < *ND4* < *COII* < *ND5* < *ND1* < *CYTB* < *ATP8* < *ND2* (Figure 3E). The Ka/Ks ratio is widely used as an indicator of selective pressure under Darwinian evolution, and it is essential to simulate evolutionary influences on a molecular scale across both homogeneous and heterogeneous species [75]. A Ka/Ks ratio greater than ‘1’ indicates positive selection, a ratio of ‘1’ suggests neutral selection, and a ratio less than ‘1’ implies negative selection [76]. The results of this study revealed that all Ka/Ks values were below 1, indicating strong negative selection among the three *Selene* species (*S. dorsalis*, *S. vomer*, and *S. setapinnis*) (Table S5). This suggests that natural selection acts to minimize harmful mutations, consistent with trends observed in various vertebrate species, including teleosts [48]. Furthermore, examining Ka/Ks ratios in the mitogenomes of *Selene* species provides valuable insights into natural selection, the evolutionary trajectory, and the dispersion of these species, helping to clarify the interplay between mutations and selective pressures and their combined effect on protein evolution.

### 3.4. Ribosomal RNA and Transfer RNA

The mitogenome of *S. dorsalis* includes two rRNA subunits: *12S rRNA* (955 bp) and *16S rRNA* (1715 bp), totaling 2670 bp, which constitutes 16.14% of the entire mitogenome. Detailed analysis revealed that the length of rRNAs varies slightly among species, ranging from 2670 bp in *S. dorsalis* to 2669 bp in *S. vomer*. Unfortunately, data for *S. setapinnis* were not available in GenBank, preventing a comparative analysis for this species. The rRNA genes exhibited an AT-bias, with values ranging from 52.28% in *S. dorsalis* to 52.34% in *S. vomer*. Additionally, AT-skew values ranged from 0.192% in *S. dorsalis* to 0.195% in *S. vomer*, while GC-skew values ranged from −0.107 in *S. dorsalis* to −0.116 in *S. vomer* (Table 2). Ribosomes are crucial for establishing the proteome of eukaryotic organisms, with ribosomal proteins assembling with rRNA during transcription. The conserved loops of rRNA and circular RNAs are vital for catalytic mechanisms involved in protein synthesis, fish immunity, and growth regulation [77]. Thus, nucleotide variations within rRNA genes of different mitogenomes could serve as genetic markers for population genetics studies [78]. The tRNAs in *S. dorsalis* total 1576 bp, representing 9.5% of the mitogenome. Comparative analysis with other *Selene* species shows varying tRNA lengths, from 1556 bp in *S. vomer* to 1415 bp in *S. setapinnis*. The AT-bias in tRNAs ranges from 55.01% in *S. dorsalis* to 55.19% in *S. setapinnis*, with AT-skew values ranging from 0.114 in *S. dorsalis* to 0.001 in *S. setapinnis* (Table 2). Most tRNAs exhibit the classical cloverleaf secondary structure, except for *tRNA Serine* (S1), which lacks the DHU-arm as reported in other studies (Figure S1) [46]. Transfer RNAs are essential for protein biosynthesis and post-transcriptional regulation in all living organisms [79]. Most anticodons for the 22 tRNAs are similar among the *Selene* species, except for the tRNA-Ile (I) in *S. vomer*, which has an anticodon of TAA instead of GAT. Similarly, *S. setapinnis* displays an anticodon of GTA, contrasting with the TGA found in *S. dorsalis* and *S. vomer* (Table S6). tRNA genes act as crucial adapters in translating DNA sequences into proteins. Additionally, variations in tRNA gene positioning and significant length heteroplasmy within the WANCY region are common in mitogenomes and provide insights into the evolutionary patterns of mitochondrial genes [80].

### 3.5. Control Regions 

**Figure 4:**
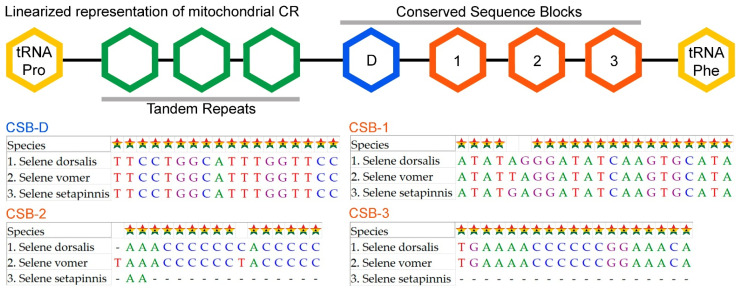
Schematic diagram comparing the length and nucleotide composition of different conserved domains in control regions of *S. dorsalis* and other *Selene* congeners. Conserved nucleotides are marked by stars.

The CR of *S. dorsalis* is 847 bp in length, accounting for 5.12% of the total mitogenome, and exhibits an AT richness of 62.81%. The CR is located between the *tRNA-Pro* (TGG) and *tRNA-Phe* (GAA) genes (Table 1). Comparative analysis of the CRs in different *Selene* species revealed variations in length, ranging from 862 bp in *S. vomer* to 718 bp in *S. setapinnis*. The AT-skew values ranged from 0.038 in *S. dorsalis*, 0.064 in *S. setapinnis*, to 0.009 in *S. vomer*. GC-skew values varied from −0.213 in *S. dorsalis*, −0.176 in *S. setapinnis*, to −0.266 in *S. vomer* (Table 2). The CR of *Selene* species includes four conserved domains: CSB-D, CSB-1, CSB-2, and CSB-3. This configuration is also found in the mitogenomes of other teleost species [81]. Within *S. dorsalis*, the conserved domains are as follows: CSB-1 has the longest base pair length (22 bp), followed by CSB-3 (19 bp), CSB-D (18 bp), and CSB-2, which has the shortest length (17 bp) (Figure 4). The AT-rich CR region has potential for assessing the population structure of the *Selene* species. The variability in nucleotide composition in this region allows for distinguishing between different populations and individuals. Additionally, the CR is crucial for regulating transcription and replication processes within the mitochondrial genome [82].

### 3.6. Phylogenetic Relationship of Carangidae

**Figure 5:**
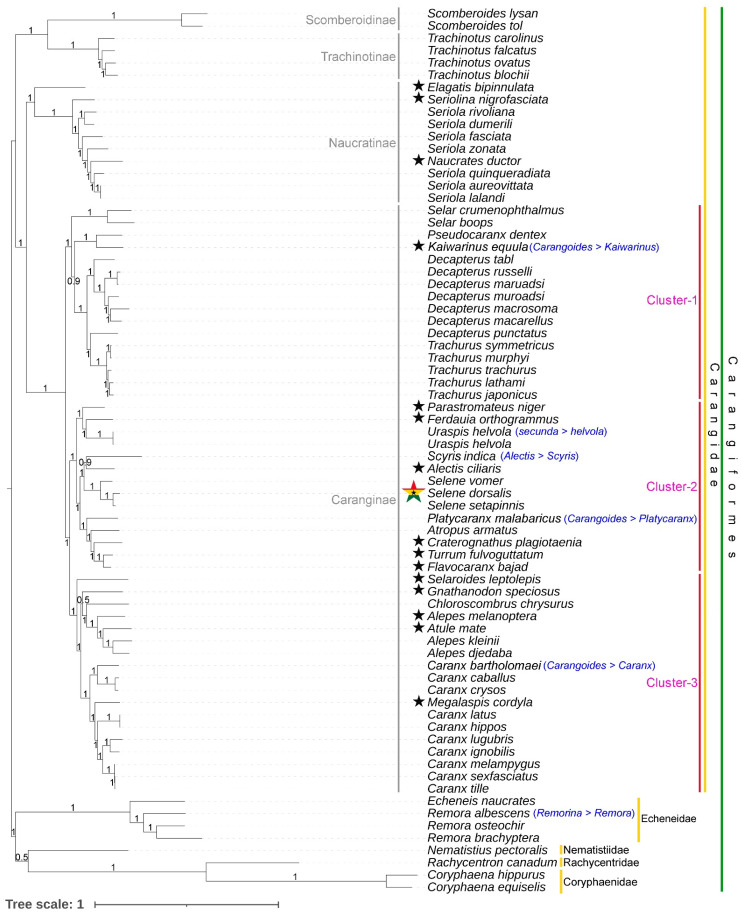
Bayesian (BA) phylogeny based on 13 concatenated PCGs, showing the differentiation of *S. dorsalis* (marked by the star-shaped map of Ghana) from other *Selene* congeners. The cladistic pattern provides insights into the evolutionary relationships at various taxonomic levels (subfamily and family) within Carangiformes. Posterior probability values at each node reflect statistical support for each branching point. Black stars indicate monotypic species within the family Carangidae. Erroneously named taxa in GenBank are indicated with blue text.

The cladistic analysis of concatenated 13 PCGs effectively delineated the Carangidae family into monophyletic clusters in both BA and ML phylogenies with high posterior probability and bootstrap support (Figure 5 and Figure S2). Members of other families, including Echeneidae, Nematistiidae, Rachycentridae, and Coryphaenidae, also formed distinct clusters within the Carangiformes phylogeny. Within Carangidae, species from the subfamilies Scomberoidinae, Trachinotinae, Naucratinae, and Caranginae displayed clear monophyletic groupings in the Bayesian and ML analysis of the mitogenome. Notably, Scomberoidinae and Trachinotinae were resolved as sister groups, while Naucratinae and Caranginae also exhibited close evolutionary relationships. Interestingly, within Caranginae, three distinct clusters were identified. In BA phylogeny, the cluster-1 includes species from the genera *Selar*, *Decapterus*, *Trachurus*, and the monotypic genus *Kaiwarinus*. The cluster-2 encompasses species from the genera *Uraspis*, *Scyris*, *Selene*, *Platycaranx*, and *Atropus*, as well as six monotypic genera (*Parastromateus*, *Ferdauia*, *Alectis*, *Craterognathus*, *Turrum*, and *Flavocaranx*). The cluster-3 includes species from the genera *Chloroscombrus*, *Alepes*, and *Caranx*, and five monotypic genera (*Selaroides*, *Gnathanodon*, *Alepes*, *Atule*, and *Megalaspis*) (Figure 5). The phylogenetic relationships observed in this study align with recent findings on the evolutionary relationships of this diverse clade of marine fishes (Carangoidei), as assessed through the capture of 1314 ultraconserved elements (UCEs) and mitogenomes [63,83,84]. The present topology also supports the revised classification at both the genus and species levels within Carangidae. However, the close association of the monotypic *Naucrates ductor* within the *Seriola* clade (subfamily Naucratinae) and the placement of the monotypic *Megalaspis cordyla* within the *Caranx* clade (subfamily Caranginae) suggest that the systematics of these species may need to be revisited. These species have broad distributions in tropical and warm-temperate seas, as well as in the Red Sea and Indo-West Pacific, respectively.

### 3.7. Lineage Diversification of Selene Species

**Figure 6:**
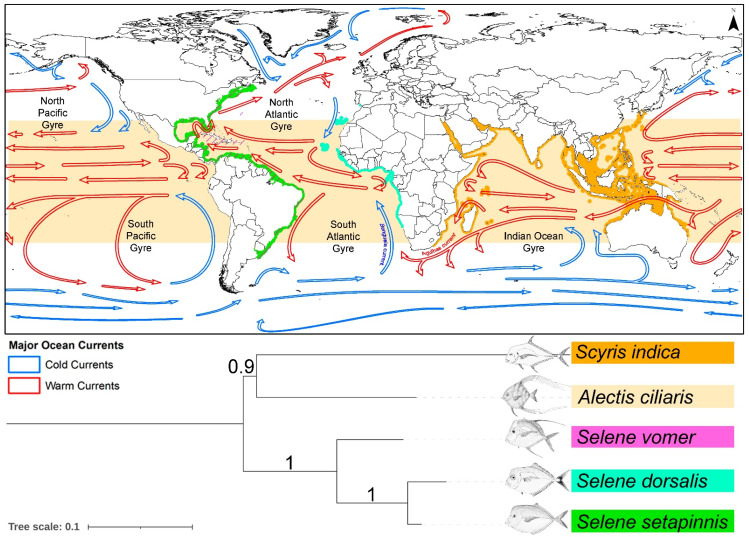
Composite figure of the pruned Bayesian tree and global range distribution illustrating the potential impact of major ocean currents on the diversification of *Selene* species in both the Eastern and Western Atlantic Oceans. Ocean currents are displayed on a global scale, compiled from the NOAA National Weather Service and the US Army (https://data.amerigeoss.org/dataset/major-ocean-currents-arrowpolys-100m-76 accessed on 15 August 2024). Illustrations of *Selene* and other carangids were sourced from Wikimedia Commons. The map was generated using ArcGIS version 10.6 and manually edited in Adobe Photoshop CS 8.0.

The suborder Carangoidei, within the order Carangiformes, encompasses a diverse range of species, including *Selene* species, which inhabit various marine environments from coral reefs to open pelagic zones [63]. Investigating the diversification and biogeographical distribution of Carangoidei provides valuable insights into marine speciation mechanisms, evolutionary history, and ecological changes [85]. The diversification of Carangoidei highlights the complex interactions between historical, ecological, and biogeographical factors that contribute to marine speciation. The Eocene epoch, marked by significant climate shifts, plate tectonic movements, and oceanographic changes, is identified as a pivotal period for the early diversification of this lineage [86,87]. The observed sympatry in Carangoidei contrasts with traditional allopatric speciation models, suggesting that ecological variation in coral reefs and habitat fragmentation play crucial roles in driving speciation within marine ecosystems [63]. Marine organisms do not distribute randomly; instead, their spatial distribution is influenced by profound biotic factors such as recruitment, competition, and predation, as well as abiotic factors like water quality and salinity, and historical factors including hurricanes and tsunamis [88,89]. Species exhibit varied responses to environmental changes, which can lead to allopatric or sympatric speciation. This mechanism is crucial for understanding the evolutionary diversity of species, particularly in marine environments [63]. Biogeographic data on *Selene* species and their cladistic patterns suggest that *S. vomer* and *S. setapinnis* share similar geographical distributions in the Western Atlantic Ocean, potentially undergoing sympatric speciation with *S. brownii*. However, due to the absence of mitogenome sequences for *S. peruviana* and *S. brevoortii*, the study could not fully explore their biogeographic patterns. These species might be separated by the Isthmus of Panama, which could contribute to allopatric speciation. Notably, the mitogenomic phylogeny reveals a close relationship between the Eastern Atlantic *S. dorsalis* and the Western Atlantic *S. setapinnis*, compared to other Western Atlantic species like *S. vomer*, consistent with recent studies [63]. The presence of an open ocean between the Eastern and Western Atlantic Oceans acts as a vicariant barrier, contributing to isolation by distance and limiting the distribution ranges of these species. Further research with detailed molecular data on additional extant species is needed to clarify their lineage diversification. Moreover, within the broader carangid phylogeny, the *Selene* lineage is closely related to the widely distributed *Alectis ciliaris* and *Scyris indica*, which are found circumglobally in tropical, subtropical, and temperate seas, as well as in the Red Sea and Indo-West Pacific, respectively (Figure 6). The cold-water barrier formed by the Benguela and Agulhas currents off the southern coast of South Africa likely separates the Atlantic populations of these carangids, promoting speciation through endemic radiation. Additionally, the unique oceanic currents in the North and South Atlantic and Pacific gyres may act as significant barriers to the diversification and adaptation of *Selene* species in these oceans (Figure 6).

### 3.8. Conservation Implication of Selene Species in the Eastern Atlantic Ocean

Global climate change is significantly affecting the ecology and geographical distribution of marine fish populations, particularly reef-associated species [90]. These changes are expected to impact marine ecosystems profoundly, leading to alterations in ocean acidity, temperature shifts, changes in food webs, shifting species distributions, rising sea levels, and reduced ocean productivity [91,92]. For carangids, such changes may lead to habitat destruction and degradation, especially in coastal, estuarine, and coral reef environments where these species are commonly found, particularly in Western Africa [93]. Ocean warming has already influenced the distribution of several carangid species, including *Seriola fasciata*, *S. dorsalis*, and *Caranx crysos*, causing them to migrate northward in search of more suitable thermal environments [12,15,16,17,18,94,95]. This migration can reduce local productivity and disrupt ecosystems and fishing communities that rely on carangids and other reef-associated fishes for economic activities, especially in West African countries [96,97]. Therefore, implementing effective fisheries management policies is crucial to ensure sustainable harvesting practices and mitigate the risk of overexploitation, which can lead to declining Catch Per Unit Effort (CPUE), particularly in Ghana where this study was conducted [98]. Additionally, employing adaptive management strategies is essential, including the use of molecular methods to accurately identify species and assess their genetic diversity. This approach will help address the impacts of climate change and ensure the long-term sustainability of carangid fisheries [99]. While this study provides insights into the *S. dorsalis* mitogenome sequence from Ghana, generating molecular data from other West African countries would offer a broader perspective on the genetic variability, population structure, and gene flow of this Atlantic moonfish. Such data are vital for the sustainable conservation of this endemic carangid species in the Eastern Atlantic.

## 4. Conclusions

The escalating impacts of global warming and extreme temperature events are threatening marine biodiversity, leading to significant declines in fisheries worldwide. Carangids, which are economically and recreationally valuable reef-associated fish, are particularly affected. Despite the importance of these species, our understanding of their evolutionary patterns, particularly through mitogenomic analysis, remains limited on a global scale. This research provides an extensive examination of the structure and variations within *Selene* mitogenomes, with a focus on the complete mitogenome of *S. dorsalis*, which is endemic to the Eastern Atlantic Ocean. The study uncovers substantial structural variations among *S. dorsalis* and its allopatric congeners, *S. setapinnis* and *S. vomer*, offering critical insights into their evolutionary dynamics. In-depth analysis of mitogenome sequences facilitates comprehensive phylogenetic investigations, elucidating the evolutionary relationships among *Selene* species and other carangid taxa. This research also enhances our understanding of how historical events and environmental factors have influenced the restricted parapatric speciation of *Selene* species in the Eastern and Western Atlantic Oceans, as well as the Eastern Pacific Ocean. These findings provide valuable resources for further studies on carangid identification, conservation genetics, speciation, and other aspects of evolutionary biology. The genetic insights gained are crucial for developing effective conservation strategies for *Selene* species, protecting species diversity, and ensuring the sustainability of marine ecosystems, particularly within the Carangidae family.

## Figures and Tables

**Table 1:** List of annotated mitochondrial genes, including their boundaries, sizes, and intergenic nucleotides (IN) for *S. dorsalis*.

Genes	Start	Stop	Size (bp)	Strand	IN	Start Codon	Stop Codon	Anticodon
*tRNA-Phe (F)*	1	68	68	+	−1	.	.	GAA
*12S rRNA*	68	1022	955	+	−1	.	.	.
*tRNA-Val (V)*	1022	1094	73	+	0	.	.	TAC
*16S rRNA*	1095	2809	1715	+	−1	.	.	.
*tRNA-Leu (L2)*	2809	2883	75	+	0	.	.	TAA
*ND1*	2884	3858	975	+	4	ATG	TAA	.
*tRNA-Ile (I)*	3863	3933	71	+	−2	.	.	GAT
*tRNA-Gln (Q)*	3932	4003	72	−	−2	.	.	TTG
*tRNA-Met (M)*	4002	4072	71	+	0	.	.	CAT
*ND2*	4073	5117	1045	+	−1	ATG	T--	.
*tRNA-Trp (W)*	5117	5188	72	+	0	.	.	TCA
*tRNA-Ala (A)*	5189	5258	70	−	0	.	.	TGC
*tRNA-Asn (N)*	5259	5332	74	−	37	.	.	GTT
*tRNA-Cys (C)*	5370	5437	68	−	−1	.	.	GCA
*tRNA-Tyr (Y)*	5437	5507	71	−	1	.	.	GTA
*COI*	5509	7059	1551	+	−1	GTG	TAA	.
*tRNA-Ser (S2)*	7059	7130	72	−	2	.	.	TGA
*tRNA-Asp (D)*	7133	7203	71	+	7	.	.	GTC
*COII*	7211	7901	691	+	−1	ATG	T--	.
*tRNA-Lys (K)*	7901	7976	76	+	1	.	.	TTT
*ATP8*	7978	8145	168	+	−10	ATG	TAA	.
*ATP6*	8136	8818	683	+	0	ATG	TA-	.
*COIII*	8819	9603	785	+	−1	ATG	TAA	.
*tRNA-Gly (G)*	9603	9673	71	+	0	.	.	TCC
*ND3*	9674	10,022	349	+	−1	ATG	T--	.
*tRNA-Arg (R)*	10,022	10,091	70	+	1	.	.	TCG
*ND4L*	10,093	10,389	297	+	−7	ATG	TAA	.
*ND4*	10,383	11,763	1381	+	−1	ATG	T--	.
*tRNA-His (H)*	11,763	11,835	73	+	−1	.	.	GTG
*tRNA-Ser (S1)*	11,835	11,903	69	+	5	.	.	GCT
*tRNA-Leu (L1)*	11,909	11,982	74	+	0	.	.	TAG
*ND5*	11,983	13,821	1839	+	−4	ATG	TAA	.
*ND6*	13,818	14,339	522	−	−1	.	.	.
*tRNA-Glu (E)*	14,339	14,408	70	−	3	.	.	TTC
*CYTB*	14,412	15,552	1141	+	−1	ATG	TAA	.
*tRNA-Thr (T)*	15,552	15,624	73	+	−2	.	.	TGT
*tRNA-Pro (P)*	15,623	15,694	72	−	0	.	.	TGG
*Control region*	15,695	16,541	847	+	.	.	.	.

**Table 2:** Nucleotide composition of mitochondrial genomes across different *Selene* species.

Species Name	Size (bp)	A%	T%	G%	C%	A + T%	AT-Skew	GC-Skew
Complete mitogenome								
*S. dorsalis*	16,541	27.48	25.66	16.69	30.18	53.13	0.034	−0.288
*S. vomer*	16,558	27.79	25.56	16.20	30.44	53.35	0.042	−0.305
PCGs								
*S. dorsalis*	11,427	25.83	26.63	15.29	32.25	52.46	−0.015	−0.357
*S. vomer*	11,428	26.29	26.33	14.77	32.61	52.62	−0.001	−0.377
*S. setapinnis*	11,427	25.11	27.04	16.12	31.73	52.15	−0.037	−0.326
tRNAs								
*S. dorsalis*	1576	30.65	24.37	19.92	25.06	55.01	0.114	−0.114
*S. vomer*	1556	30.59	24.23	20.18	25.00	54.82	0.116	−0.107
*S. setapinnis*	1415	27.63	27.56	23.89	20.92	55.19	0.001	0.066
rRNAs								
*S. dorsalis*	2670	31.161	21.12	21.31	26.40	52.28	0.192	−0.107
*S. vomer*	2669	31.285	21.06	21.06	26.60	52.34	0.195	−0.116
CRs								
*S. dorsalis*	847	32.59	30.22	14.64	22.55	62.81	0.038	−0.213
*S. vomer*	862	32.60	32.02	12.99	22.39	64.62	0.009	−0.266
*S. setapinnis*	718	34.68	30.50	14.35	20.47	65.18	0.064	−0.176

## Data Availability

The genome sequence data that support the findings of this study are openly available in GenBank of NCBI at https://www.ncbi.nlm.nih.gov, under the accession no. PP857611.

## Supplementary Materials

The following supporting information can be downloaded at https://www.mdpi.com/article/10.3390/biom14101208/s1, Figure S1. Secondary structures of 22 transfer RNAs (tRNAs) of *Selene dorsalis* display the structural variation. The tRNAs are denoted by full names and IUPAC-IUB single-letter amino acid codes. The first structure shows the nucleotide positions and details of the stem–loop of tRNAs. Watson–Crick and wobble base pairing are marked by black and red color bars, respectively; Figure S2. Maximum-likelihood (ML) phylogeny based on 13 concatenated PCGs, showing the cladistic relationship of Carangiformes including *Selene* congeners; Table S1. Details of the mitogenomes of Carangiformes (family Carangidae, Echeneidae Coryphaenidae Rachycentridae, and Nematistiidae) species acquired from the GenBank phylogenetic analyses; Table S2. Comparison of intergenic nucleotides of three different *Selene* species mitogenomes; Table S3. Comprehensive comparison of the start and stop codons of the PCGs across three *Selene* mitogenomes; Table S4. The abundance of amino acids and RSCU value of the complete PCGs of three *Selene* species; Table S5. Comparative pairwise Ka/Ks values of each PCG for three *Selene* species; Table S6. Detailed comparison of anticodons found in the transfer RNA genes within three *Selene* mitogenomes.
