# Supplementary material for: Endemic Radiation of African Moonfish, Selene dorsalis (Gill 1863), in the Eastern Atlantic: Mitogenomic Characterization and Phylogenetic Implications of Carangids (Teleostei: Carangiformes)
Source: Biomolecules. 2024 Sep 25;14(10):1208. doi: 10.3390/biom14101208 (PMC11506752; doi:10.3390/biom14101208)
Supplement: Supplementary file 1 [file biomolecules-14-01208-s001.zip › biomolecules-3194978-supplementary.pdf]

Supplementary Materials

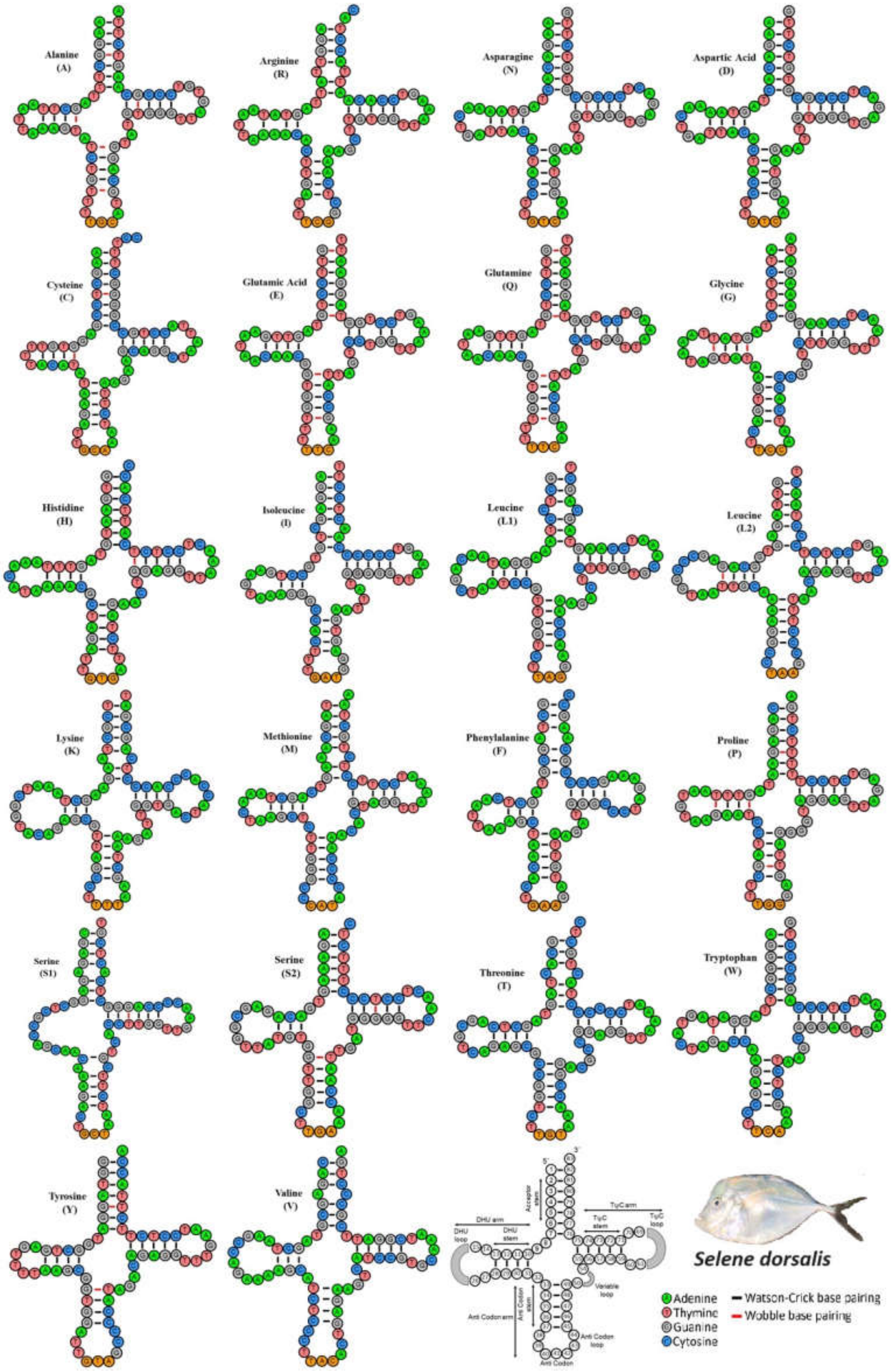

Figure S1. Secondary structures of 22 transfer RNAs (tRNAs) of *Selene dorsalis* display the structural variation. The tRNAs are denoted by full names and IUPAC-IUB single-letter amino acid codes. The first structure shows the nucleotide positions and details of the stem–loop of tRNAs. Watson–Crick and wobble base pairing are marked by black and red color bars, respectively.

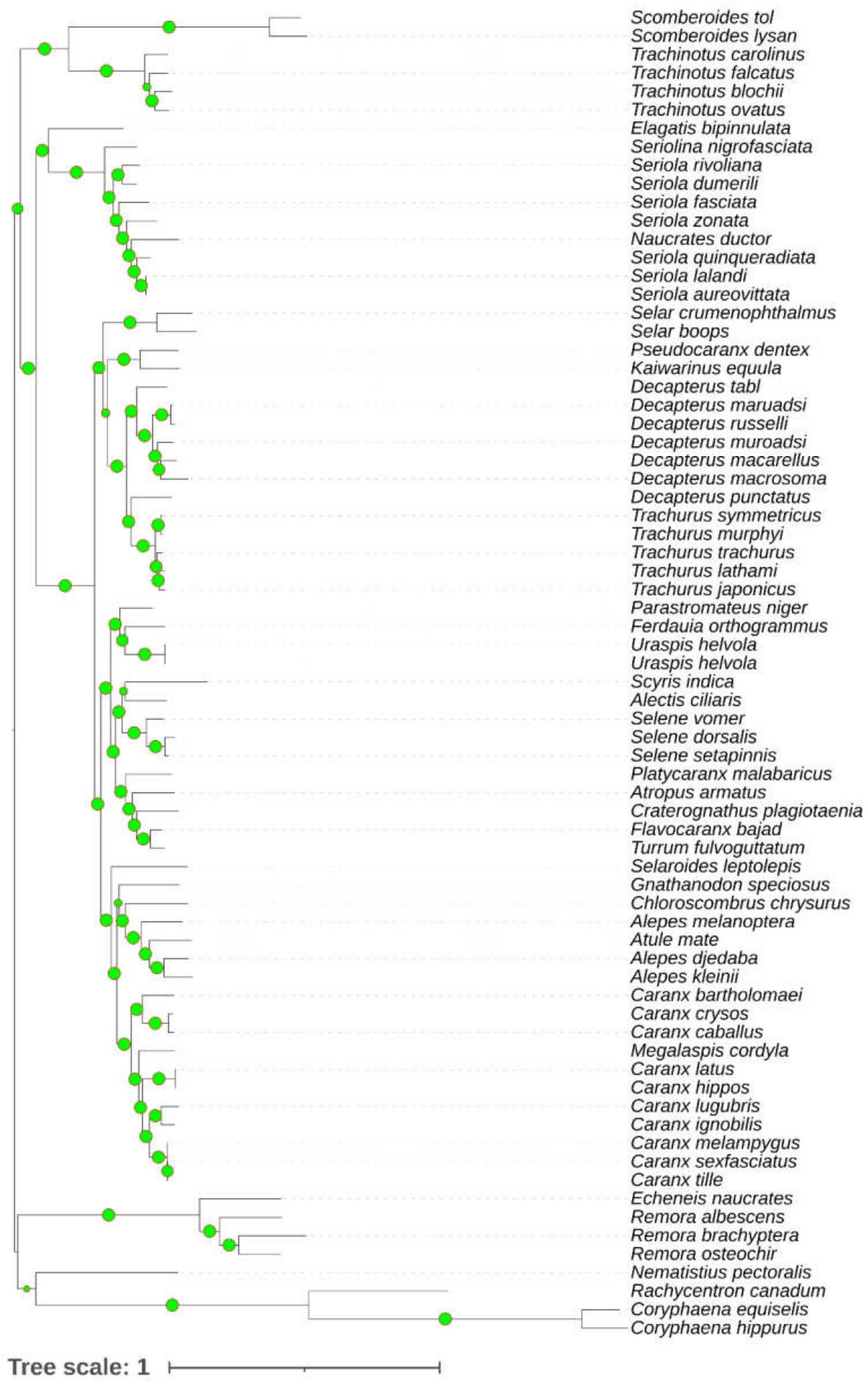

Figure S2. Maximum-likelihood (ML) phylogeny based on 13 concatenated PCGs, showing the cladistic relationship of Carangiformes including *Selene* congeners.

Table S1. Details of the mitogenomes of Carangiformes (family Carangidae, Echeneidae Coryphaenidae Rachycentridae, and Nematistiidae) species acquired from the GenBank phylogenetic analyses.

Sl. No.	Family	GenBank Name	Valid Name	Accession Number	References
1	Carangidae	*Carangoides bartholomaei*	*Caranx bartholomaei*	PP032955	[45]
2	Carangidae	*Carangoides malabaricus*	*Platycaranx malabaricus*	KJ174514	Li et al., 2016c
3	Carangidae	*Carangoides equula*	*Kaiwarinus equula*	KM201334	Zou & Li, 2016
4	Carangidae	*Uraspis helvola*	*Uraspis helvola*	KM978993	Unpublished
5	Carangidae	*Uraspis secunda*	*Uraspis helvola*	KT819204	Ma et al., 2017b
6	Carangidae	*Parastromateus niger*	*Parastromateus niger*	KJ192332	Chen et al., 2016
7	Carangidae	*Alectis ciliaris*	*Alectis ciliaris*	KM522837	Li et al., 2016f
8	Carangidae	*Selene vomer*	*Selene vomer*	PP033011	[45]
9	Carangidae	*Selene setapinnis*	*Selene setapinnis*	OR575618	[44]
10	Carangidae	*Selene dorsalis*	*Selene dorsalis*	PP857611	This Study
11	Carangidae	*Atropus armatus*	*Atropus armatus*	AP004444	[8]
12	Carangidae	*Atule mate*	*Atule mate*	KM522838	Li et al., 2014
13	Carangidae	*Alepes melanoptera*	*Alepes melanoptera*	PP231954	Unpublished
14	Carangidae	*Alepes djedaba*	*Alepes djedaba*	KP408222	[73]
15	Carangidae	*Alepes kleinii*	*Alepes kleinii*	KF728081	Li & Zou, 2016
16	Carangidae	*Gnathanodon speciosus*	*Gnathanodon speciosus*	MT922005	Jiang et al., 2022
17	Carangidae	*Selaroides leptolepis*	*Selaroides leptolepis*	OP546178	Halasan et al., 2024
18	Carangidae	*Chloroscombrus chrysurus*	*Chloroscombrus chrysurus*	PP032962	[45]
19	Carangidae	*Caranx crysos*	*Caranx crysos*	MW435597	Jeon et al., 2021
20	Carangidae	*Caranx tille*	*Caranx tille*	KT805946	Ma et al., 2016
21	Carangidae	*Caranx ignobilis*	*Caranx ignobilis*	KF649842	Santos et al., 2011
22	Carangidae	*Caranx melampygus*	*Caranx melampygus*	AP004445	[8]
23	Carangidae	*Caranx hippos*	*Caranx hippos*	OP035266	[44]
24	Carangidae	*Caranx latus*	*Caranx latus*	PP032957	[45]
25	Carangidae	*Caranx caballus*	*Caranx caballus*	OP057043	[44]
26	Carangidae	*Caranx lugubris*	*Caranx lugubris*	OP035166	[44]
27	Carangidae	*Caranx sexfasciatus*	*Caranx sexfasciatus*	OP035090	[44]
28	Carangidae	*Craterognathus plagiotaenia*	*Craterognathus plagiotaenia*	MT677872	Kaure et al., 2021
29	Carangidae	*Megalaspis cordyla*	*Megalaspis cordyla*	KM522836	Li et al., 2016d
30	Carangidae	*Trachurus trachurus*	*Trachurus trachurus*	AB108498	Takashima et al., 2006
31	Carangidae	*Trachurus murphyi*	*Trachurus murphyi*	PP533446	Asorey et al., 2014
32	Carangidae	*Trachurus japonicus*	*Trachurus japonicus*	AP003092	Unpublished
33	Carangidae	*Trachurus lathami*	*Trachurus lathami*	OP057107	[44]
34	Carangidae	*Trachurus symmetricus*	*Trachurus symmetricus*	OR482443	[44]
35	Carangidae	*Decapterus muroadsi*	*Decapterus muroadsi*	OP057048	[44]
36	Carangidae	*Decapterus maruadsi*	*Decapterus maruadsi*	KJ004518	Li et al., 2016a
37	Carangidae	*Decapterus russelli*	*Decapterus russelli*	MN711693	[82]
38	Carangidae	*Decapterus tabl*	*Decapterus tabl*	MN102718	[73]
39	Carangidae	*Decapterus macarellus*	*Decapterus macarellus*	KM986880	Zou et al., 2016

40	Carangidae	*Decapterus macrosoma*	*Decapterus macrosoma*	KF841444	Li et al., 2016e
41	Carangidae	*Decapterus punctatus*	*Decapterus punctatus*	OP035108	[44]
42	Carangidae	*Selar boops*	*Selar boops*	MW581014	Unpublished
43	Carangidae	*Selar crumenophthalmus*	*Selar crumenophthalmus*	KJ148633	Li et al., 2016b
44	Carangidae	*Pseudocaranx dentex*	*Pseudocaranx dentex*	MZ359280	[83]
45	Carangidae	*Naucrates ductor*	*Naucrates ductor*	OR546204	[44]
46	Carangidae	*Seriola dumerili*	*Seriola dumerili*	MZ398238	Unpublished
47	Carangidae	*Seriola lalandi*	*Seriola lalandi*	OR482442	[44]
48	Carangidae	*Seriola quinqueradiata*	*Seriola quinqueradiata*	AB517556	Iguchi et al., 2012
49	Carangidae	*Seriola rivoliana*	*Seriola rivoliana*	AB517559	Iguchi et al., 2012
50	Carangidae	*Seriola zonata*	*Seriola zonata*	OP035199	[44]
51	Carangidae	*Seriola fasciata*	*Seriola fasciata*	OR482462	[44]
52	Carangidae	*Seriola aureovittata*	*Seriola aureovittata*	MH211123	Shi et al., 2019
53	Carangidae	*Seriolina nigrofasciata*	*Seriolina nigrofasciata*	KT591876	Liu et al., 2016
54	Carangidae	*Elagatis bipinnulata*	*Elagatis bipinnulata*	KT824759	Ma et al., 2017a
55	Carangidae	*Trachinotus carolinus*	*Trachinotus carolinus*	KJ556976	Zhang et al., 2016
56	Carangidae	*Trachinotus blochii*	*Trachinotus blochii*	KJ184305	Zhang et al., 2014
57	Carangidae	*Trachinotus ovatus*	*Trachinotus ovatus*	KF356397	Xie et al., 2015
58	Carangidae	*Trachinotus falcatus*	*Trachinotus falcatus*	OP056886	[44]
59	Carangidae	*Alectis indica*	*Scyris indica*	KP710215	[73]
60	Carangidae	*Scomberoides tol*	*Scomberoides tol*	OR668917	Unpublished
61	Carangidae	*Scomberoides lysan*	*Scomberoides lysan*	MZ329991	[44]
62	Carangidae	*Flavocaranx bajad*	*Flavocaranx bajad*	LC557137	Unpublished
63	Carangidae	*Ferdauia orthogrammus*	*Ferdauia orthogrammus*	OP035068	[44]
64	Carangidae	*Turrum fulvoguttatum*	*Turrum fulvoguttatum*	OR499749	[44]
65	Echeneidae	*Remora brachyptera*	*Remora brachyptera*	OR546234	[44]
66	Echeneidae	*Remora osteochir*	*Remora osteochir*	OR575559	[44]
67	Echeneidae	*Echeneis naucrates*	*Echeneis naucrates*	AB355905	Miya et al., 2013
68	Echeneidae	*Remorina albescens*	*Remora albescens*	OP057074	[44]
69	Coryphaenidae	*Coryphaena equiselis*	*Coryphaena equiselis*	PP032965	[45]
70	Coryphaenidae	*Coryphaena hippurus*	*Coryphaena hippurus*	MH576915	Xu et al., 2018
71	Rachycentridae	*Rachycentron canadum*	*Rachycentron canadum*	FJ154956	Wang et al., 2011
72	Nematistiidae	*Nematistius pectoralis*	*Nematistius pectoralis*	ON838225	[47]

Supporting References:

Asorey, C.; Larraín, M.; Araneda, C. The Complete Mitochondrial Genome of the Chilean Jack Mackerel, *Trachurus murphyi* Nichols, 1920 (Teleostei, Carangidae). **2024**, Proceedings of [Congreso de Ciencias del Mar 2024 at: Talcahuano, Chile].

Chen, H.; Xie, Z.; Huang, H.; Yang, Z.; Zhang, Y.; Zhang, J.; Li, S.; Zhang, Y. The complete mitochondrial genome of the *Parastromateus niger* (Perciformes, Carangidae). *Mitochondrial DNA A DNA Mapp Seq Anal.* **2016**, 27, 332–334.

Halasan, L.C.; Lin, H.C. Diagnostic Applicability of Mitogenomics in Uncovering Intraspecific Carangid Diversifications: Insights into Phylogeny, Divergence Time, and Characterization of Two Cryptic *Selaroides leptolepis* Mitogenomes. *Org. Divers. Evol.* **2024**, *1-18*.

Iguchi, J.; Takashima, Y.; Namikoshi, A.; Ueno, K.; Hara, T. Species identification method for marine products of *Seriola* and related species. *Fish. Sci*. **2012**, 78, 197–206.

Jeon, A.Y.; Lee, J.H.; Andriyono, S.; Zuweh, J.A.; Kim, H.W. The complete mitochondrial genome of the blue runner, *Caranx crysos* (Mitchill, 1815) (Teleostei: Carangidae). *Mitochondrial DNA B Resour.* **2021**, 6, 1519–1520.

Jiang, F.; Yang, N.; Huang, H. Complete mitochondrial DNA sequence of Golden trevally *Gnathanodon speciosus* (Forsskål, 1775) and the phylogenetic analysis of Carangidae. *Mitochondrial DNA B Resour*. **2022**, 7, 1321–1322.

Kaure, T.; Tabassum, N.; Kim, H.; Kim, Y.M.; Kim, H.W. The Complete Mitochondrial Genome of the Barcheek Trevally, *Carangoides plagiotaenia* Bleeker, 1857 from Beqa Lagoon in Fiji. *Mitochondrial DNA B Resour.* **2021**, *6*, 1810–1811.

Li, M.; Chen, Z.; Chen, T.; Xiong, D.; Fan, J.; Liang, P. Whole mitogenome of the Japanese scad *Decapterus maruadsi* (Perciformes: Carangidae*). Mitochondrial DNA A DNA Mapp Seq Anal.* **2016a**, 27, 306–307.

Li, M.; Chen, Z.; Zou, K. Complete mitochondrial genome of the bigeye scad *Selar crumenophthalmus* (Perciformes: Carangidae). *Mitochondrial DNA A DNA Mapp Seq Anal.* **2016b**, 27, 308–309.

Li, M.; Huang, Z.; Chen, Z. Characterization of the mitochondrial genome of the Malabar trevally *Carangoides malabaricus* and related phylogenetic analyses. *Mitochondrial DNA A DNA Mapp Seq Anal.* **2016c**, 27, 378–379.

Li, M.; Li, Y.; Chen, Z. Description of the Mitochondrial Genome of Yellowtail Scad *Atule mate* (Perciformes: Carangidae). *Mitochondrial DNA A DNA Mapp Seq Anal.* **2014**, *27*, 2186–2187.

Li, M.; Li, Y.; Chen, Z. Mitochondrial Genome of the Torpedo Scad *Megalaspis cordyla* (Perciformes: Carangidae): Genome Characterization and Phylogenetic Consideration. *Mitochondrial DNA A DNA Mapp Seq Anal.* **2016d**, *27*, 1856–1857.

Li, M.; Zhang, P.; Sun, D.; Chen, T.; Fan, J.; Zou, K.; Chen, Z. Characterization of the mitochondrial genome of the Shortfin scad *Decapterus macrosoma* (Perciformes: Carangidae). *Mitochondrial DNA A DNA Mapp Seq Anal.* **2016e**, 27, 82–83.

Li, M.; Zou, K. Organization and characterization of the mitochondrial genome of the Razorbelly scad *Alepes kleinii* (Perciformes: Carangidae). *Mitochondrial DNA A DNA Mapp Seq Anal.* **2016**, 27, 100–101.

Li, Y.; Chen, Z.; Zhang, P.; Liang, P.; Chen, S.; Guo, Y.; Li, M. Complete mitochondrial genome of the African pompano *Alectis ciliaris* (Perciformes: Carangidae). *Mitochondrial DNA A DNA Mapp Seq Anal.* **2016f**, 27, 1858–1859.

Liu, J.; Kong, L.; Gao, Y.; Wu, C. Complete mitochondrial genome of black-banded trevally (*Seriolina nigrofasciata*): Genome characterization and phylogenetic considerations. *Mitochondrial DNA A DNA Mapp Seq Anal.* **2016**, 27, 4701–4703.

Ma, C.; Ma, H.; Zhang, H.; Feng, C.; Wei, H.; Wang, W.; Chen, W.; Zhang, F.; Ma, L. The complete mitochondrial genome sequence and gene organization of the rainbow runner (*Elagatis bipinnulata*) (Perciformes: Carangidae). *Mitochondrial DNA A DNA Mapp Seq Anal.* **2017a**, 28, 5–6.

Ma, H.; Ma, C.; Zhang, H.; Zhang, X.; Feng, C.; Zhang, Y.; Wang, W.; Chen, W.; Ma, L. Characterization of the complete mitochondrial genome and phylogenetic relationship of *Caranx tille* (Perciformes: Carangidae). *Mitochondrial DNA A DNA Mapp Seq Anal.* **2016**, 27, 4704–4705.

Ma, H.; Ma, C.; Zhang, X.; Feng, C.; Zhang, Y.; Zhang, H.; Ma, L. The first complete mitochondrial genome sequence of *Uraspis secunda* (Perciformes: Carangidae) and its phylogenetic relationship. *Mitochondrial DNA A DNA Mapp Seq Anal.* **2017b**, 28, 87–88.

Miya, M.; Friedman, M.; Satoh, T.P.; Takeshima, H.; Sado, T.; Iwasaki, W.; Yamanoue, Y.; Nakatani, M.; Mabuchi, K.; Inoue, J.G.; Poulsen, J.Y.; Fukunaga, T.; Sato, Y.; Nishida, M. Evolutionary Origin of the Scombridae (Tunas and Mackerels): Members of a Paleogene Adaptive Radiation with 14 Other Pelagic Fish Families. *PloS ONE* **2013**, *8*, e73535.

Santos, S.R.; Xiang, Y.; Tagawa, A.W. Population Structure and Comparative Phylogeography of Jack Species (*Caranx ignobilis* and *C. melampygus*) in the High Hawaiian Islands. *J. Hered.* **2011**, *102*, 47-54.

Shi, B.; Liu, X.; Xu, Y.; Wang, B.; Jiang, Y. Complete Mitochondrial Genome of Yellowtail Kingfish *Seriola aureovittata* (Perciformes, Carangidae). *Mitochondrial DNA B Resour.* **2019**, *4*, 517–518.

Takashima, Y.; Morita, T.; Yamashita, M. Complete mitochondrial DNA sequence of Atlantic horse mackerel *Trachurus trachurus* and molecular identification of two commercially important species *T. trachurus* and *T. japonicus* using PCR-RFLP. *Fish. Sci.* **2006**, 72, 1054–1065.

Wang, Z.; Guo, Y.; Liu, C.; Liu, Y. The Complete Mitochondrial DNA of Cobia (*Rachycentron canadum*) and Phylogenetics of Carangoid. *Acta Hydrobiol. Sin.* **2011**,35, 229-237.

Xie, Z.; Li, S.; Yao, M.; Lu, D.; Li, Z.; Meng, Z.; Zhang, Y.; Lin, H. The complete mitochondrial genome of the *Trachinotus ovatus* (Teleostei, Carangidae). *Mitochondrial DNA* **2015**, 26, 644–646.

Xu, L.; Wang, X.; Li, H.; Du, F. Complete Mitogenome of Two Dolphinfishes (*Coryphaena hippurus* and *Coryphaena equiselis*) from South China Sea. *Mitochondrial DNA B Resour.* **2018**, *3*, 1098–1099.

Zhang, D.; Wang, L.; Guo, H.; Ma, Z.; Jiang, S. The Complete Mitochondrial Genome of Snubnose Pompano *Trachinotus blochii* (Teleostei, Carangidae). *Mitochondrial DNA A DNA Mapp Seq Anal.* **2014**, *27*, 431–432.

Zhang, D.; Wang, L.; Guo, H.; Ma, Z.; Zhang, N.; Lin, J.; Jiang, S. Complete mitochondrial genome of Florida pompano *Trachinotus carolinus* (Teleostei, Carangidae). *Mitochondrial DNA A DNA Mapp Seq Anal.* **2016**, 27, 597–598.

Zou, K.; Chen, Z.; Zhang, P.; Li, M. Mitochondrial Genome of the Mackerel Scad *Decapterus macarellus* (Perciformes: Carangidae). *Mitochondrial DNA A DNA Mapp Seq Anal.* **2016**, *27*, 2151–2152.

Zou, K.; Li, M. Characterization of the mitochondrial genome of the Whitefin trevally *Carangoides equula* (Perciformes: Carangidae): A novel initiation codon for ATP6 gene. *Mitochondrial DNA A DNA Mapp Seq Anal.* **2016**, 27, 1779–1780.

Table S2. Comparison of intergenic nucleotides of three different *Selene* species mitogenomes.

Genes	*Selene dorsalis*	*Selene vomer*	*Selene setapinnis*
tRNA-Phe (F)	-1	0	-1
12S rRNA	-1	0	-1
tRNA-Val (V)	0	0	0
16S rRNA	-1	0	0
tRNA-Leu (L2)	0	0	0
ND1	4	5	5
tRNA-Ile (I)	-2	-1	-1
tRNA-Gln (Q)	-2	-1	-1
tRNA-Met (M)	0	0	0
ND2	-1	0	0
tRNA-Trp (W)	0	1	1
tRNA-Ala (A)	0	1	1
tRNA-Asn (N)	37	38	38
tRNA-Cys (C)	-1	0	0
tRNA-Tyr (Y)	1	1	1
COI	-1	0	0
tRNA-Ser (S2)	2	3	3
tRNA-Asp (D)	7	7	7
COII	-1	0	0
tRNA-Lys (K)	1	1	1
ATP8	-10	-10	-10
ATP6	0	-1	-1
COIII	-1	0	0
tRNA-Gly (G)	0	0	0
ND3	-1	0	0
tRNA-Arg (R)	1	1	1
ND4L	-7	-7	-7
ND4	-1	0	0
tRNA-His (H)	-1	0	0
tRNA-Ser (S1)	5	6	6
tRNA-Leu (L1)	0	0	0
ND5	-4	-4	-4
ND6	-1	0	0
tRNA-Glu (E)	3	4	3
Cyt b	-1	0	0
tRNA-Thr (T)	-2	-1	-1
tRNA-Pro (P)	0	0	-1
Control region	-	-	-

Table S3. Comprehensive comparison of the start and stop codons of the PCGs across three *Selene* mitogenomes.

**Genes**	***Selene dorsalis***		***Selene vomer***		***Selene setapinnis***	
	Start	Stop	Start	Stop	Start	Stop
ND1	ATG	TAA	ATG	TAA	ATG	TAA
ND2	ATG	T--	ATG	T--	ATG	T--
COI	GTG	TAA	GTG	TAA	GTG	TAA
COII	ATG	T--	ATG	T--	ATG	T--
ATP8	ATG	TAA	ATG	TAA	ATG	TAA
ATP6	ATG	TA-	ATG	TAA	ATG	TAA
COIII	ATG	TA-	ATG	TA-	ATG	TA-
ND3	ATG	T--	ATG	T--	ATG	T--
ND4L	ATG	TAA	ATG	TAA	ATG	TAA
ND4	ATG	T--	ATG	T--	ATG	T--
ND5	ATG	TAA	ATG	TAG	ATG	TAG
ND6	ATG	TAG	ATG	TAG	ATG	TAG
Cyt b	ATG	T--	ATG	T--	ATG	T--

Table S4. The abundance of amino acids and RSCU value of the complete PCGs of three *Selene* species.

Selene dorsalis											
Codon	Count	RSCU	Codon	Count	RSCU	Codon	Count	RSCU	Codon	Count	RSCU
UUU(F)	91	1.11	UCU(S)	72	1.06	UAU(Y)	102	1.16	UGU(C)	24	0.62
UUC(F)	73	0.89	UCC(S)	108	1.59	UAC(Y)	74	0.84	UGC(C)	54	1.38
UUA(L)	76	0.77	UCA(S)	58	0.85	UAA(*)	85	1.55	UGA(W)	66	1.26
UUG(L)	36	0.37	UCG(S)	32	0.47	UAG(*)	26	0.47	UGG(W)	39	0.74
CUU(L)	142	1.44	CCU(P)	159	1.42	CAU(H)	78	0.93	CGU(R)	41	0.94
CUC(L)	164	1.66	CCC(P)	157	1.4	CAC(H)	90	1.07	CGC(R)	53	1.21
CUA(L)	110	1.12	CCA(P)	94	0.84	CAA(Q)	90	1.4	CGA(R)	34	0.78
CUG(L)	63	0.64	CCG(P)	39	0.35	CAG(Q)	39	0.6	CGG(R)	47	1.07
AUU(I)	98	1.22	ACU(T)	77	1.05	AAU(N)	110	1.1	AGU(S)	50	0.74
AUC(I)	63	0.78	ACC(T)	93	1.27	AAC(N)	90	0.9	AGC(S)	88	1.29
AUA(M)	67	1.21	ACA(T)	94	1.28	AAA(K)	84	1.4	AGA(*)	61	1.11
AUG(M)	44	0.79	ACG(T)	29	0.4	AAG(K)	36	0.6	AGG(*)	47	0.86
GUU(V)	36	1.37	GCU(A)	44	0.9	GAU(D)	23	0.87	GGU(G)	33	0.87
GUC(V)	30	1.14	GCC(A)	88	1.81	GAC(D)	30	1.13	GGC(G)	48	1.27
GUA(V)	27	1.03	GCA(A)	45	0.92	GAA(E)	59	1.48	GGA(G)	39	1.03
GUG(V)	12	0.46	GCG(A)	18	0.37	GAG(E)	21	0.53	GGG(G)	31	0.82
Selene vomer											
Codon	Count	RSCU	Codon	Count	RSCU	Codon	Count	RSCU	Codon	Count	RSCU
UUU(F)	74	0.98	UCU(S)	80	1.23	UAU(Y)	86	1.14	UGU(C)	25	0.67
UUC(F)	77	1.02	UCC(S)	98	1.51	UAC(Y)	65	0.86	UGC(C)	50	1.33
UUA(L)	85	0.9	UCA(S)	54	0.83	UAA(*)	57	1.23	UGA(W)	72	1.24
UUG(L)	37	0.39	UCG(S)	34	0.52	UAG(*)	30	0.65	UGG(W)	44	0.76
CUU(L)	148	1.56	CCU(P)	157	1.54	CAU(H)	75	0.99	CGU(R)	38	0.9
CUC(L)	149	1.57	CCC(P)	141	1.38	CAC(H)	77	1.01	CGC(R)	61	1.45
CUA(L)	93	0.98	CCA(P)	85	0.83	CAA(Q)	80	1.4	CGA(R)	28	0.67
CUG(L)	57	0.6	CCG(P)	26	0.25	CAG(Q)	34	0.6	CGG(R)	41	0.98
AUU(I)	86	1.11	ACU(T)	70	1.12	AAU(N)	91	1.03	AGU(S)	56	0.86
AUC(I)	69	0.89	ACC(T)	82	1.32	AAC(N)	86	0.97	AGC(S)	68	1.05
AUA(M)	49	1.01	ACA(T)	69	1.11	AAA(K)	77	1.48	AGA(*)	52	1.12
AUG(M)	48	0.99	ACG(T)	28	0.45	AAG(K)	27	0.52	AGG(*)	46	0.99
GUU(V)	28	1.22	GCU(A)	36	0.8	GAU(D)	31	1	GGU(G)	24	0.69
GUC(V)	28	1.22	GCC(A)	87	1.92	GAC(D)	31	1	GGC(G)	57	1.64
GUA(V)	21	0.91	GCA(A)	47	1.04	GAA(E)	44	1.35	GGA(G)	34	0.98
GUG(V)	15	0.65	GCG(A)	11	0.24	GAG(E)	21	0.65	GGG(G)	24	0.69
Selene setapinnis											
Codon	Count	RSCU	Codon	Count	RSCU	Codon	Count	RSCU	Codon	Count	RSCU
UUU(F)	87	1.12	UCU(S)	77	1.21	UAU(Y)	83	1.12	UGU(C)	29	0.67
UUC(F)	69	0.88	UCC(S)	101	1.59	UAC(Y)	65	0.88	UGC(C)	58	1.33
UUA(L)	74	0.77	UCA(S)	45	0.71	UAA(*)	64	1.35	UGA(W)	62	1.18
UUG(L)	36	0.38	UCG(S)	31	0.49	UAG(*)	31	0.65	UGG(W)	43	0.82
CUU(L)	138	1.45	CCU(P)	155	1.52	CAU(H)	74	0.94	CGU(R)	39	0.92
CUC(L)	159	1.66	CCC(P)	131	1.29	CAC(H)	84	1.06	CGC(R)	50	1.18
CUA(L)	106	1.11	CCA(P)	84	0.83	CAA(Q)	76	1.39	CGA(R)	37	0.88
CUG(L)	60	0.63	CCG(P)	37	0.36	CAG(Q)	33	0.61	CGG(R)	43	1.02
AUU(I)	87	1.18	ACU(T)	76	1.18	AAU(N)	95	1.14	AGU(S)	52	0.82
AUC(I)	61	0.82	ACC(T)	78	1.21	AAC(N)	72	0.86	AGC(S)	76	1.19
AUA(M)	57	1.21	ACA(T)	71	1.1	AAA(K)	68	1.43	AGA(*)	48	1.01
AUG(M)	37	0.79	ACG(T)	33	0.51	AAG(K)	27	0.57	AGG(*)	47	0.99
GUU(V)	35	1.46	GCU(A)	36	0.8	GAU(D)	29	1	GGU(G)	36	0.95
GUC(V)	27	1.13	GCC(A)	83	1.83	GAC(D)	29	1	GGC(G)	45	1.18
GUA(V)	18	0.75	GCA(A)	40	0.88	GAA(E)	46	1.35	GGA(G)	40	1.05
GUG(V)	16	0.67	GCG(A)	22	0.49	GAG(E)	22	0.65	GGG(G)	31	0.82

Table S5. Comparative pairwise Ka/Ks values of each PCG for three *Selene* species.

Species	*nad1*	*nad2*	*cox1*	*cox2*	*atp8*	*atp6*	*cox3*	*nad3*	*nad4l*	*nad4*	*nad5*	*nad6*	*Cytb*
S. vomer vs *S. setapinnis*	0.0083	0.0319	0.0043	0.0141	0.0314	0.0114	0.0050	0.0000	0.0000	0.0124	0.0000	0.0089	0.0171
S. vomer vs *S. dorsalis*	0.0121	0.0325	0.0050	0.0137	0.0344	0.0110	0.0048	0.0000	0.0000	0.0120	0.0181	0.0098	0.0130
S. setapinnis vs *S. dorsalis*	0.0252	0.0329	0.0000	0.0000	0.0000	0.0000	0.0000	0.0000	0.0000	0.0000	0.0181	0.0000	0.0297
Average	0.0152	0.0324	0.0031	0.0093	0.0219	0.0075	0.0033	0.0000	0.0000	0.0081	0.0121	0.0062	0.0199
STDEV	0.0088	0.0005	0.0027	0.0080	0.0191	0.0065	0.0028	0.0000	0.0000	0.0070	0.0104	0.0054	0.0087

Table S6. Detailed comparison of anticodons found in the transfer RNA genes within three *Selene* mitogenomes.

Genes	*Selene dorsalis*	*Selene vomer*	*Selene setapinnis*
tRNA-Phe (F)	GAA	GAA	-
tRNA-Val (V)	TAC	TAC	-
tRNA-Leu (L2)	TAA	TAA	TAA
tRNA-Ile (I)	GAT	TAA	GAT
tRNA-Gln (Q)	TTG	TTG	TTG
tRNA-Met (M)	CAT	CAT	CAT
tRNA-Trp (W)	TCA	TCA	TCA
tRNA-Ala (A)	TGC	TGC	TGC
tRNA-Asn (N)	GTT	GTT	GTT
tRNA-Cys (C)	GCA	GCA	GCA
tRNA-Tyr (Y)	GTA	GTA	GTA
tRNA-Ser (S2)	TGA	TGA	GTA
tRNA-Asp (D)	GTC	GTC	GTC
tRNA-Lys (K)	TTT	TTT	TTT
tRNA-Gly (G)	TCC	TCC	TCC
tRNA-Arg (R)	TCG	TCG	TCG
tRNA-His (H)	GTG	GTG	GTG
tRNA-Ser (S1)	GCT	GCT	GCT
tRNA-Leu (L1)	TAG	TAG	TAG
tRNA-Glu (E)	TTC	TTC	TTC
tRNA-Thr (T)	TGT	TGT	TGT
tRNA-Pro (P)	TGG	TGG	TGG
